# The impact of negative COVID-19 experiences on cancer survivors’ health-related quality of life and psychological distress: a moderated mediation model

**DOI:** 10.3389/fpsyg.2024.1423106

**Published:** 2024-09-12

**Authors:** Blanca S. Noriega Esquives, Akina Natori, Michael H. Antoni, Amy K. Otto, Sarah Prinsloo, Richard W. Wagner, Telma I. Gomez, Cassandra A. Hathaway, Cornelia M. Ulrich, Anita R. Peoples, Lorenzo Cohen, Frank J. Penedo

**Affiliations:** ^1^Sylvester Comprehensive Cancer Center, Miller School of Medicine, University of Miami, Miami, FL, United States; ^2^Division of Medical Oncology, Department of Medicine, Miller School of Medicine, University of Miami, Miami, FL, United States; ^3^Department of Psychiatry and Behavioral Sciences, Miller School of Medicine, University of Miami, Miami, FL, United States; ^4^University of Minnesota Medical School, Duluth, MN, United States; ^5^Department of Palliative, Rehabilitation and Integrative Medicine, The University of Texas M. D. Anderson Cancer Center, Houston, TX, United States; ^6^Department of Neurosurgery, The University of Texas M. D. Anderson Cancer Center, Houston, TX, United States; ^7^Department of Pediatrics Hematology-Oncology, Baylor College of Medicine, Houston, TX, United States; ^8^Westat, Rockville, MD, United States; ^9^Huntsman Cancer Institute, University of Utah, Salt Lake City, UT, United States; ^10^Department of Population Health Sciences, University of Utah, Salt Lake City, UT, United States; ^11^Department of Psychology, University of Miami, Miami, FL, United States; ^12^Department of Medicine, Miller School of Medicine, University of Miami, Miami, FL, United States

**Keywords:** cancer survivors, COVID-19 pandemic, financial hardship, health-related quality of life, moderated mediation analysis, psychological distress

## Abstract

**Introduction:**

Cancer survivors experienced poorer health-related quality of life (HRQoL) and greater psychological distress during the COVID-19 pandemic than those without cancer. However, the underlying mechanisms that may explain how negative experiences during the pandemic are associated with distress and HRQoL remain unknown. We examined whether psychosocial risk factors (i.e., healthcare disruption, disruption to daily activities and social interaction [DDASI], and financial hardship) mediated the relationship between negative COVID-19-related experiences and cancer survivors’ HRQoL and psychological distress (i.e., depressive symptoms, and anxiety) and whether the mediating effects were moderated by psychosocial protective factors (i.e., stress management ability and social support).

**Methods:**

A total of 9,651 cancer survivors completed a questionnaire assessing negative COVID-19-related experiences, psychosocial and practical experiences, and HRQoL. Conditional process analysis was used to evaluate the proposed moderated mediation models.

**Results:**

Participants had a mean age of 63.8 years (SD = 12.3) and were mostly non-Hispanic White (82.3%). DDASI and financial hardship mediated the relationship between negative COVID-19-related experiences and cancer survivor’s HRQoL and psychological distress. Stress management ability buffered the indirect effect of DDASI on cancer survivors’ HRQoL and psychological distress. Social support buffered the indirect effect of financial hardship on HRQoL and depressive symptoms.

**Conclusion:**

Financial resources and social interactions may buffer negative effects of major disruptions such as the COVID-19 pandemic. Future studies should assess the longitudinal impact of these associations.

## 1 Introduction

The COVID-19 pandemic was one of the most challenging and disruptive global events experienced in several generations. While the federal COVID-19 Public Health Emergency declaration ended on May 11, 2023, the COVID-19 pandemic presented a chronic and challenging public health threat that continues to impact all communities (Nelson and Kaminsky, 2020). Cancer survivors are considered particularly vulnerable due to their increased propensity to infections and poor immunological responses to COVID-19 vaccines and boosters, as well as ongoing cancer-related stressors (Becerril-Gaitan et al., 2022; Ligumsky et al., 2022; Naranbhai et al., 2022). One of the challenges among cancer survivors regarding the COVID-19 pandemic is the trade-off between increased risk of contracting COVID-19 while receiving treatment/follow-up care vs. reduced risk of COVID-19 infection by postponing treatment/follow-up (Burki, 2020). As cancer survivors are already coping with multiple, and often chronic, challenges associated with a cancer diagnosis and treatment, these were exacerbated by the COVID-19 pandemic (Ayubi et al., 2021; Momenimovahed et al., 2021; Muls et al., 2022). These challenges include, but are not limited to, emotional and social concerns regarding disease activity, treatment response, interpersonal disruptions, healthcare disruptions, and financial burden (Steel et al., 2022; Williams et al., 2022). Collectively, these chronic stressors can significantly further impact health-related quality of life (HRQoL) and psychological distress, such as symptoms of anxiety and depression. However, the characterization of potential processes by which pandemics may impact psychological distress and HRQoL in cancer survivors remains relatively unknown.

A robust psychosocial literature has documented the role of multiple psychosocial factors on HRQoL and psychological distress outcomes in cancer populations. These include concerns about unmet needs specific to health care delivery, daily activities, social interaction, and finances (Brose et al., 2023; Ghazal et al., 2023; Lu et al., 2023; Tan et al., 2023). Furthermore, the impact of these factors on adverse outcomes can vary according to socio-economic status (SES) and other social determinants of health (Bambra et al., 2020; Chu et al., 2022). Multiple studies have reported worse HRQoL and greater emotional distress among cancer survivors during the COVID-19 pandemic; however, limited research has examined the underlying mechanisms that may explain how negative experiences during the COVID-19 pandemic may impact psychological distress and HRQoL outcomes (Ayubi et al., 2021; Muls et al., 2022). Conversely, resilience factors such as social support, perceived benefits in the face of challenges, and stress management skills have been shown to favorably impact HRQoL and other patient-reported outcomes (Velasco-Durantez et al., 2022; Guidry et al., 2023). Identifying factors associated with psychological distress and HRQoL is the first step in mitigating the deleterious effects of added stressors on cancer survivors by intervening on the modifiable risk and protective factors.

**Figure 1:**
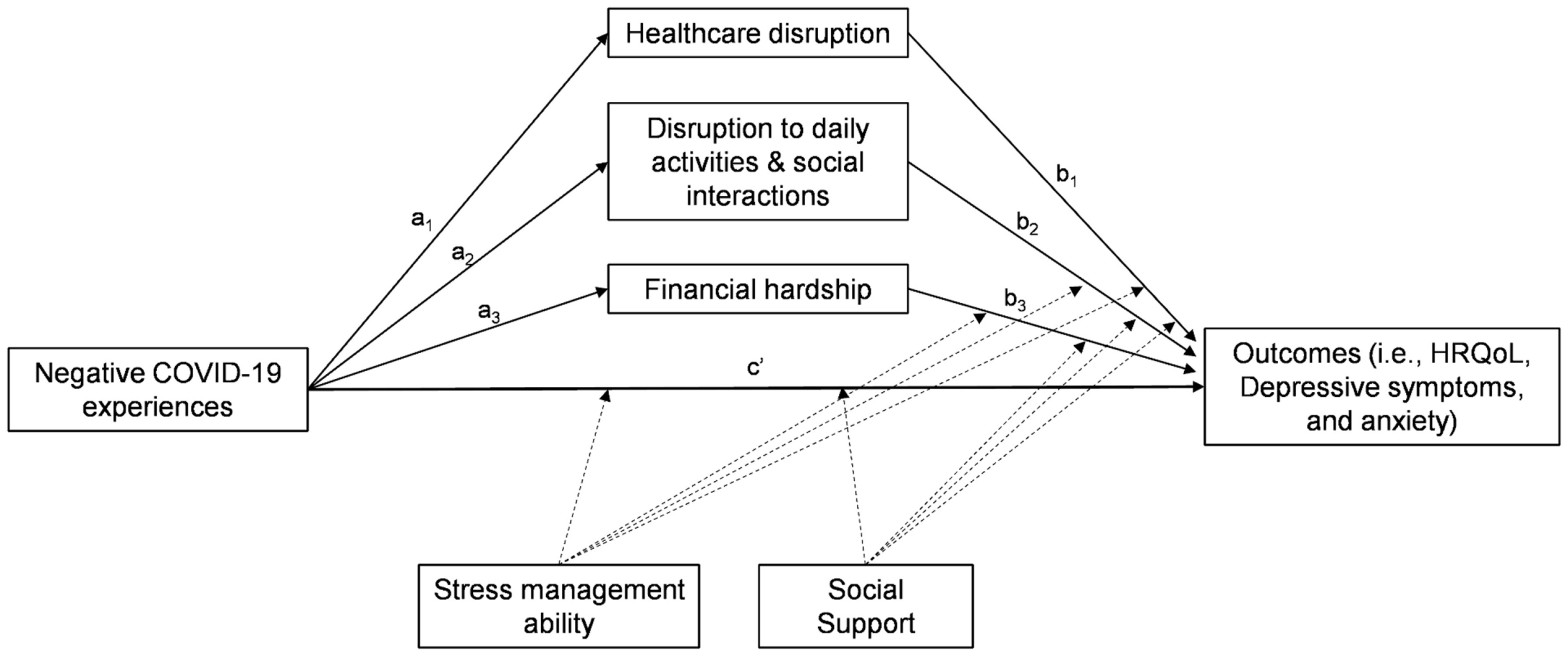
Conceptual moderated mediation model. HRQoL, Health-related quality of life.

This study aimed to:(1) assess whether psychosocial risk factors [i.e., healthcare disruption, disruption to daily activities and social interaction (DDASI), and financial hardship] mediated the relationship between negative COVID-19-related experiences and cancer survivors’ HRQoL and psychological distress (i.e., anxiety and depressive symptoms), and (2) assess whether psychosocial protective factors (i.e., stress management ability and social support) moderated any mediating effect of psychosocial risk factors in the relationship between negative COVID-19-related experiences and cancer survivors’ HRQoL and psychological distress. We thus examined the following hypotheses: (1) greater negative COVID-19-related experiences would be associated with poorer HRQoL and greater psychological distress and psychosocial risk factors; (2) the relationship between negative COVID-19-related experiences and cancer survivors’ HRQoL and psychological distress would be mediated by psychosocial risk factors, and (3) psychosocial protective factors would moderate the relationship between negative COVID-19-related experiences and cancer survivors’ HRQoL and psychological distress such that at higher levels of psychosocial protective factors the direct effect of negative COVID-19-related experiences as well as the indirect effects of psychosocial risk factors become weaker (Figure 1).

## 2 Materials and methods

### 2.1 Participants

This study involved an online survey conducted between May 2020 and January 2021 to investigate the impact of the COVID-19 pandemic on cancer survivors. An individual is considered a cancer survivor from the time of diagnosis through the balance of life (National Cancer Institute, 2024). Participants were recruited from the University of Texas MD Anderson Cancer Center (MDACC; IRB #2020-0508) and the University of Miami Sylvester Comprehensive Cancer Center (SCCC; IRB #202000450). Inclusion criteria were: ≥18 years old; English- or Spanish-speaking; MDACC or SCCC patient; attended a clinical visit within the past 5 years; ICD-10 confirmed cancer diagnosis; and contactable via email or electronic patient portal. Patients received either an email or patient portal message with the link to the online REDCap survey (Harris et al., 2009; Harris et al., 2019). All participants signed an electronic informed consent prior to data collection.

### 2.2 Measures

#### 2.2.1 Negative COVID-19-related experiences

Survey participants were asked whether or not they underwent adverse COVID-19 experiences. This questionnaire included 21 questions and were answered as Yes = 1 or No = 0. Sample items include: “If you tested positive for COVID-19, were you hospitalized?,” “Did a family member or a member of your household die of COVID-19?,” “Did you lose your job or primary source of income due to COVID-19?” The summation of the responses represented the total number of negative COVID-19-related experiences (Perry et al., 2023).

#### 2.2.2 COVID-19-related psychosocial and practical experiences questionnaire

The COVID-PPE is a validated questionnaire that assesses COVID-19-related psychological distress (i.e., anxiety and depressive symptoms) and psychosocial risk and protective factors (Saez-Clarke et al., 2023). This 5-point Likert scale instrument, ranging from 0 (strongly disagree) to 4 (strongly agree), includes 43 items and yields nine subscales (i.e., COVID-19 specific anxiety and depressive symptoms, healthcare disruption, satisfaction with provider response, disruption to daily activities and social interaction, financial hardship, perceived benefit, social support, and perceived stress management ability). A subscale score was estimated by averaging the corresponding items (range 0–4), with a higher score indicating a higher level of the construct being assessed (Cronbach’s alphas [α’s] = 0.61–0.89). The correlations across the psychosocial risk factors range from 0.29 to 0.56. The correlation between the psychosocial protective factors was 0.37. See Saez-Clarke et al. (2023) for additional details on the development and psychometric evaluation of the COVID-PPE questionnaire.

#### 2.2.3 Health-related quality of life

HRQoL was measured using the Functional Assessment of Cancer Therapy-General-7 Item Version (FACT-G7), a well-validated measure of HRQoL in oncology (Pearman et al., 2014). Four item scores were reverse-coded. The total score ranges from 0 to 28, with a higher total score indicating better HRQoL (α = 0.80).

#### 2.2.4 Sociodemographic and clinical characteristics

Age, sex, self-reported race and ethnicity, insurance type (i.e., managed care, Medicaid, Medicare, other/self-pay), cancer diagnosis and stage (metastatic vs. non-metastatic disease), and ZIP code of residence were extracted from electronic health records. SES was assessed with the area deprivation index (ADI), which is a validated multidimensional measure of neighborhood socioeconomic disadvantages based on 17 census indicators assessing education, income, household characteristics, and housing (Kind and Buckingham, 2018). ADI ranges from 1, least deprived, to 100, the most deprived neighborhood.

### 2.3 Data analysis

The survey responses were inspected for data quality check. Descriptive analysis was computed for all variables to ensure values were within expected ranges and errors were eliminated. Then, descriptive statistics (i.e., mean, standard deviation, counts, and percentages) were calculated using SAS version 9.4 (SAS Institute, Cary, NC). Moderated mediation analysis was conducted with the PROCESS macro for SAS version 4.3.1 (Copyright 2017–2023 by Andrew F. Hayes), a regression-based modeling tool for mediation, moderation, and conditional process analysis (Hayes and Rockwood, 2020; Hayes, 2022). First, we estimated separate parallel multiple mediation models to assess the impact of negative COVID-19-related experiences (X) on each outcome (i.e., HRQoL, anxiety symptoms, and depressive symptoms). The involved mediators of the models were: healthcare disruption (M1), disruption to daily activities and social interaction (M2), and financial hardship (M3). To examine whether stress management ability (W1) and social support (W2) moderated the direct effect of negative COVID-19-related experiences as well as the specific indirect effect of each mediator, eight interaction terms were included in the moderated mediation models (e.g., X*W1, X*W2, M1*W1, M1*W2). “High” and “low” levels of both moderators were determined at one standard deviation above and below the mean of each scale. Bias-corrected bootstrapping methods with 5,000 bootstrap samples were used to examine the significance of the effects, which were considered significant if the resulting confidence interval did not contain 0. Continuous variables were grand mean centered and categorial covariates were dummy coded. To account for potential confounding by sociodemographic and medical variables, all models were adjusted for study site, age, sex, race/ethnicity, ADI score, insurance type, cancer type (hematologic vs. solid tumor), and whether patients had metastatic disease. We computed the variance inflation factor (VIF) of each variable to assess the degree of multicollinearity. All VIFs were below two suggesting that the model estimations did not have a multicollinearity bias (Noora, 2020). The effects of the covariates were expressed in terms of standardized regression coefficients (betas).

## 3 Results

A total of 11,325 patients responded to the questionnaire. Of these, 9,651 cancer survivors had valid mailing addresses and were included in this study. Overall, participants had a mean age of 63.8 years (SD = 12.3). The majority were female (57.3%), non-Hispanic White (82.3%), and had managed care (50.3%) or Medicare (45.8%). Participants had a mean ADI score of 39.6 (SD = 24.5). The majority were diagnosed with a solid tumor (84.5%) and had non-metastatic disease (77.3%). Regarding psychosocial characteristics, out of the 21 possible negative COVID-19-related experiences, participants reported a mean of 7.3 negative experiences (SD = 1.9). From the COVID-PPE subscale scores (range 0–4), mean depressive symptoms score was 1.75 (SD = 0.98); anxiety symptoms score was 2.33 (SD = 0.92); healthcare disruption was 1.76 (SD = 1.13); disruption to daily activities and social interaction was 2.24 (SD = 0.86); financial hardship was 1.09 (SD = 0.85); perceived stress management skills was 2.67 (SD = 0.60); and social support was 2.60 (SD = 0.63). They also had a mean FACT-G7 score of 19.0 (SD = 5.3; Supplementary Table S1).

### 3.1 Sociodemographic and clinical characteristics and study outcomes

Tables 1–3 present the effects of the covariates on both study outcomes and mediators. Older age was associated with greater HRQoL, and less depressive and anxiety symptoms, and psychosocial risk factors (i.e., healthcare disruption, DDASI, financial hardship). Compared to men, women reported worse HRQoL, and more depressive and anxiety symptoms, and psychosocial risk factors. Compared to non-Hispanic Whites, non-Hispanic Black survivors had reported less healthcare disruption, but more anxiety symptoms. Hispanic cancer survivors reported more anxiety symptoms and financial hardship than non-Hispanic Whites. Cancer survivors with a higher ADI (more socioeconomically disadvantaged) had worse HRQoL, fewer depressive symptoms, less disruption to daily activities and social interaction, and more financial hardship. Compared to survivors with solid tumors, those with hematologic cancers reported worse HRQoL and more anxiety symptoms and disruption to daily activities and social interaction. Survivors with metastatic disease had worse HRQoL and more anxiety symptoms compared to those with stage 0-III disease.

**Table 1:** Moderated mediation model of HRQOL.

Regression coefficients				
Predictors	Outcome	Mediators
	HRQOL	HCD	DDASI	FH
Intercept	23.055**	2.177**	2.565**	1.636**
Exposure				
Negative COVID-19 experiences (NCE)	−0.032	0.005	0.042**	0.057**
Covariates				
Age	0.019*	−0.004*	−0.005**	−0.010**
Gender, female	−0.820**	0.099*	0.208**	0.057*
Race/Ethnicity				
Non-Hispanic Black	−0.208	−0.190*	−0.087	0.078
Non-Hispanic, other race	0.577*	0.149*	0.004	0.148*
Hispanic	0.061	−0.038	0.055	0.204**
Area deprivation index	−0.007*	0.001	−0.002**	0.002**
Insurance type				
Managed care	0.818*	−0.264*	−0.103*	−0.102*
Medicaid/Medicare	0.183	−0.161*	−0.049	−0.090
Cancer type, hematologic	−0.715**	−0.028	0.090*	0.001
Cancer stage, metastatic	−1.193**	0.001	0.021	0.006
Study Center, MD Anderson	0.501	−0.046	−0.303**	−0.390**
Mediators				
Healthcare disruption (HCD)	−0.489*	-	-	-
Disruption to daily activities (DDASI)	−3.100**	-	-	-
Financial hardship (FH)	−1.705**	-	-	-
Moderators and interaction terms				
Stress management ability (SMA)	0.472	-	-	-
NCE* SMA	−0.024	-	-	-
HCD* SMA	0.036	-	-	-
DDASI*SMA	0.712**	-	-	-
FH*SMA	−0.053	-	-	-
Social support (SS)	0.181	-	-	-
NCE* SS	0.005	-	-	-
HCD* SS	−0.084	-	-	-
DDASI*SS	−0.220*	-	-	-
FH*SS	0.310*	-	-	-
Indices of partial moderated mediation				
	Index	SE	LLCI	ULCI
Healthcare disruption				
Stress management ability	0.000	0.001	−0.001	0.002
Social support	0.000	0.001	−0.003	0.001
Disruption to daily activities				
Stress management ability	**0.030**	0.006	0.020	0.043
Social support	**−0.009**	0.005	−0.020	0.000
Financial hardship				
Stress management ability	−0.003	0.006	−0.015	0.009
Social support	**0.018**	0.007	0.005	0.031

Bold font indicates statistical significance. *p < 0.05; **p < 0.001. 95% Bootstrap Confidence Intervals. Reference group: Gender: Male; Race/Ethnicity: Non-Hispanic White; Insurance Type: Other/Self pay; Cancer Type: Solid tumor; Cancer stage: Non-metastatic (Stages 0-III); Study Center: Miami.

**Table 2:** Moderated mediation model of depressive symptoms.

Regression coefficients				
Predictors	Outcome	Mediators
	Depressive symptoms	HCD	DDASI	FH
Intercept	0.798*	2.162**	2.574**	1.622**
Exposure				
Negative COVID-19 experiences (NCE)	−0.035	0.007	0.045**	0.057**
Covariates				
Age	−0.005**	−0.004*	−0.005**	−0.010**
Gender, female	0.307**	0.100*	0.209**	0.059*
Race/Ethnicity				
Non-Hispanic Black	−0.019	−0.190*	−0.092*	0.076
Non-Hispanic, other race	−0.051	0.160*	0.005	0.148*
Hispanic	0.028	−0.038	0.032	0.179**
Area deprivation index	−0.001**	0.001	−0.002**	0.002**
Insurance type				
Managed care	−0.063	−0.250*	−0.099	−0.090
Medicaid/Medicare	−0.012	−0.151*	−0.040	−0.083
Cancer type, hematologic	0.042	−0.029	0.089	0.004
Cancer stage, metastatic	−0.033	0.000	0.020	0.007
Study Center, MD Anderson	−0.180*	−0.057	−0.322**	−0.400**
Mediators				
Healthcare disruption (HCD)	0.100*	-	-	-
Disruption to daily activities (DDASI)	0.740**	-	-	-
Financial hardship (FH)	0.284**	-	-	-
Moderators and interaction terms				
Stress management ability (SMA)	−0.100	-	-	-
NCE* SMA	0.016*	-	-	-
HCD* SMA	−0.019	-	-	-
DDASI*SMA	−0.119**	-	-	-
FH*SMA	0.042*	-	-	-
Social support (SS)	0.042	-	-	-
NCE* SS	0.000	-	-	-
HCD* SS	0.019	-	-	-
DDASI*SS	0.015	-	-	-
FH*SS	−0.070**	-	-	-
Indices of partial moderated mediation				
	Index	SE	LLCI	ULCI
Healthcare disruption				
Stress management ability	0.000	0.000	−0.001	0.000
Social support	0.000	0.000	0.000	0.001
Disruption to daily activities				
Stress management ability	**−0.005**	0.001	−0.007	−0.003
Social support	0.001	0.001	−0.001	0.002
Financial hardship				
Stress management ability	**0.002**	0.001	0.000	0.005
Social support	**−0.004**	0.001	−0.006	−0.002

Bold font indicates statistical significance. *p < 0.05; **p < 0.001. 95% Bootstrap confidence intervals. Reference group: GENDER: male; race/ethnicity: Non-Hispanic White; insurance type: other/self pay; cancer type: solid tumor; cancer stage: non-metastatic (Stages 0–III); Study Center: Miami.

**Table 3:** Moderated mediation model of anxiety.

Regression coefficients				
Predictors	Outcome	Mediators
	Anxiety	HCD	DDASI	FH
Intercept	0.922**	2.161**	2.574**	1.622**
Exposure				
Negative COVID-19 experiences (NCE)	−0.040	0.007	0.045**	0.057**
Covariates				
Age	−0.004**	−0.004*	−0.005**	−0.010**
Gender, female	0.149**	0.100*	0.209**	0.059*
Race/ethnicity				
Non-Hispanic Black	0.113*	−0.190*	−0.092*	0.076
Non-Hispanic, other race	0.230**	0.160*	0.005	0.148*
Hispanic	0.295**	−0.038	0.032	0.179**
Area deprivation index	0.000	0.001	−0.002*	0.002**
Insurance type				
Managed care	0.045	−0.250*	−0.099	−0.090
Medicaid/Medicare	0.098*	−0.151*	−0.040	−0.083
Cancer type, hematologic	0.126**	−0.029	0.089*	0.004
Cancer stage, metastatic	0.041*	0.000	0.020	0.007
Study Center, MD Anderson	−0.087	−0.057	−0.322**	−0.400**
Mediators				
Healthcare disruption (HCD)	0.142*	-	-	-
Disruption to daily activities (DDASI)	0.578**	-	-	-
Financial hardship (FH)	0.058	-	-	-
Moderators and interaction terms				
Stress management ability (SMA)	0.062	-	-	-
NCE* SMA	0.014	-	-	-
HCD* SMA	−0.014	-	-	-
DDASI*SMA	−0.065*	-	-	-
FH*SMA	0.037*	-	-	-
Social support (SS)	0.112	-	-	-
NCE* SS	0.000	-	-	-
HCD* SS	0.009	-	-	-
DDASI*SS	−0.021	-	-	-
FH*SS	−0.019	-	-	-
Indices of partial moderated mediation				
	Index	SE	LLCI	ULCI
Healthcare disruption				
Stress management ability	0.000	0.000	−0.001	0.000
Social support	0.000	0.000	−0.000	0.001
Disruption to daily activities				
Stress management ability	**−0.003**	0.001	−0.005	−0.001
Social support	−0.001	0.001	−0.003	0.001
Financial hardship				
Stress management ability	0.002	0.001	0.000	0.005
Social support	−0.001	0.001	−0.003	0.001

Bold font indicates statistical significance. *p < 0.05; **p < 0.001. 95% Bootstrap confidence intervals. Reference group: gender: male; race/ethnicity: Non-Hispanic White; insurance type: other/self pay; cancer type: solid tumor; Cancer stage: non-metastatic (Stages 0–III); Study Center: Miami.

### 3.2 Health-related quality of life

The parallel mediation model shows that the direct effect (DE) of negative COVID-19-related experiences on HRQoL was statistically significant (β = −0.083 95% Bootstrap Confidence Intervals [BCI]: −0.139, −0.028; Supplementary Table S2). The total indirect effect of the set of proposed mediators was statistically significant (β = −0.149; 95%BCI: −0.180, −0.116). Specifically, disruption to daily activities and social interaction (Indirect effect [IE] = −0.077, 95%BCI: −0.097, −0.057) and financial hardship (IE = −0.068, 95%BCI: −0.083, −0.054) mediated the relationship between negative COVID-19-related experiences and HRQoL.

The moderated mediation model shows that the DE of negative COVID-19-related experiences on HRQoL was not moderated by the proposed moderators (Table 1). Stress management ability moderated the IE of disruption to daily activities and social interaction. The interaction term was significant (β = 0.712, SE = 0.100, *p* < 0.001), and confirmed by a formal test using the index of moderated mediation [IMM] (0.030; 95%BCI: 0.020, 0.043), indicating that stress management ability buffered the IE of disruption to daily activities and social interaction on HRQoL (Supplementary Table S3; Supplementary Figure S1a). Social support moderated the IEs of disruption to daily activities and social interaction and financial hardship. The interaction terms were statistically significant (DDASI: β = −0.220, SE = 0.098, *p* = 0.023; FH: β = 0.310, SE = 0.098, *p* = 0.002), and confirmed by the IMM (DDASI: −0.009; 95%BCI: −0.020, −0.0004; FH: 0.018; 95%BCI: 0.005, 0.031). Social support enhanced the IE of disruption to daily activities and social interaction but buffered the IE of financial hardship on HRQoL (Supplementary Figure S1).

### 3.3 Depressive symptoms

The parallel mediation model shows that the total IE of the set of proposed mediators was statistically significant (β = 0.035; 95%BCI: 0.028, 0.042; Supplementary Table S4). Disruption to daily activities and social interaction (IE = 0.021, 95%BCI: 0.016, 0.026) and financial hardship (IE = 0.013, 95%BCI: 0.011, 0.016) mediated the relationship between negative COVID-19-related experiences and depressive symptoms.

The moderated mediation model shows that stress management ability moderated the IE of disruption to daily activities and social interaction and financial hardship (Table 2). The interaction terms were statistically significant (DDASI: β = −0.119, SE = 0.018, *p* < 0.0001; FH: β = 0.042, SE = 0.017, *p* = 0.012), and confirmed by the IMM (DDASI: −0.005; 95%BCI: −0.007, −0.003; FH: 0.002; 95%BCI: 0.0001, 0.005). Stress management ability buffered the IE of disruption to daily activities and social interaction but enhanced the IE of financial hardship on depressive symptoms (Supplementary Table S5; Supplementary Figure S2a). Social support moderated the IE of financial hardship on depressive symptoms. The interaction term was statistically significant (β = −0.070, SE = 0.017, *p* < 0.0001; IMM = −0.004; 95%BCI: −0.006, −0.002), indicating that social support buffered the IE of financial hardship on depressive symptoms.

### 3.4 Anxiety symptoms

The parallel mediation model shows that the total IE of the set of proposed mediators was statistically significant (β = 0.023; 95%BCI: 0.018, 0.028; Supplementary Table S6). Disruption to daily activities and social interaction (IE = 0.016, 95%BCI: 0.012, 0.020) and financial hardship (IE = 0.006, 95%BCI: 0.004, 0.008) mediated the relationship between negative COVID-19-related experiences and anxiety symptoms.

The moderated mediation model shows that stress management ability moderated the IE of disruption to daily activities and social interaction (Table 3). The interaction term was statistically significant (β = −0.065, SE = 0.018, *p* = 0.0003; IMM = -0.003; 95%BCI: −0.005, −0.001). Stress management ability buffered the IE of disruption to daily activities and social interaction on anxiety symptoms (Supplementary Table S7; Supplementary Figure S2a). Social support did not moderate any indirect effect.

## 4 Discussion

In early 2020, governments started to implement different public health measures ranging from physical distancing to full lockdowns to mitigate the spread of SARS-CoV-2. While the containment measures helped reduce COVID-19 spread, these events might also have had unintended adverse secondary effects negatively impacting on physical and mental health, causing financial hardship, and disrupting social interaction, employment, and medical care. In this study, we found that disruption to daily activities and social interaction, as well as financial hardship, mediated the relationship between negative COVID-19-related experiences and cancer survivors’ HRQoL and psychological distress. The results are consistent with prior research that reported negative impacts of the COVID-19 pandemic on psychosocial well-being and HRQoL in cancer survivors. For instance, a meta-analysis of studies examining mental health conditions among survivors during the pandemic revealed elevated levels of anxiety and depression (Ayubi et al., 2021). Furthermore, several studies showed the association between high financial burden and worse HRQoL among cancer survivors during the pandemic (Ayubi et al., 2021; Chen et al., 2021; Momenimovahed et al., 2021; Staehler et al., 2021; Muls et al., 2022). The current study contributes to our understanding of how major disruptive events, such as the COVID-19 pandemic, impact psychological distress and HRQoL in cancer survivors, by identifying potential intervention targets that may buffer the adverse effects of these acute and chronic global stressors.

Social support buffered the negative effect of financial hardship on HRQoL and depressive symptoms, while perceived stress management skills buffered the effect of disruption to daily activities on HRQoL, depressive and anxiety symptoms. Our findings align with previous research demonstrating a positive association between social support and HRQoL of cancer survivors (Gudina et al., 2021). Participants in our study may have benefitted from tangible or material support from friends and family during the COVID-19 pandemic, which likely provided some relief from financial difficulties. In addition, our results support research indicating that stress management interventions can improve cancer survivors’ psychological and physiological adaptation as well as clinical health outcomes (Antoni et al., 2023). Stress management skills may equip cancer survivors with coping mechanisms to handle disruptions (e.g., relaxation exercises, mindfulness), while also building resilience, fostering a positive outlook, and helping them focus on what can be controlled. Our findings underscore the importance of fostering strong social support networks and stress management skills to enhance the well-being of cancer survivors, particularly during times of crisis. Contrary to expectations, we also found that perceived stress management enhanced the negative effect of financial hardship on depression and social support enhanced the negative effect of disruption to daily activities and social interaction on HRQoL. Stress management skills may amplify the negative effects of financial hardship on depression by increasing awareness of financial difficulties, and promoting unrealistic expectations that these skills could mitigate completely the impact of financial strain. The latter finding, while seemingly contradictory, may indicate that cancer survivors who rely heavily on social support had greater sensitivity to disruptions in daily life and social interactions because they are accustomed to a certain level of assistance and stability. Future research should explore the nuanced roles of social support and stress management skills in different contexts to better understand their complex interactions and impacts on cancer survivors’ well-being.

Our findings showed that older cancer survivors reported less disruption to daily activities, less depressive and anxiety symptoms, and greater HRQoL. This finding is consistent with prior studies examining the impact of the COVID-19 pandemic on cancer survivors’ distress (Jeppesen et al., 2021; Koinig et al., 2021). Older survivors may have already become accustomed to restrictions in their daily life, and thus, they might be able to cope better with the additional restrictions imposed by the COVID-19 pandemic. Similar to previous studies, we also found that female cancer survivors reported worse HRQoL, greater psychosocial risk, and more depressive and anxiety symptoms (Jeppesen et al., 2021; Koinig et al., 2021). Compared to non-Hispanic Whites, Hispanics and non-Hispanic Blacks reported more anxiety symptoms, which is not surprising given racial-ethnic disparities in psychological distress prior to and during the COVID-19 pandemic (Wen et al., 2023).

Some work has documented that prevalence and mortality of COVID-19 infection were higher in traditionally vulnerable communities (Bambra et al., 2020; Maroko et al., 2020; Dioun et al., 2023). Although cancer survivors in high ADI (more disadvantaged area) reported statistically worse HRQoL, more depressive symptoms and financial hardship, and less disruption to daily life and social interaction, it should be noted that the magnitude of these differences were relatively small compared to other predictors. There is established evidence indicating that neighborhood disadvantage contributes to inequity in health outcomes through access to resources, environmental exposures, health behaviors, material deprivation, and psychological mechanisms among cancer patients (Chu et al., 2022). However, specific to subjective measures such as financial toxicity, several studies reported no difference in financial toxicities among cancer patients by SES (Esselen et al., 2023; Kieran et al., 2023). Although economic hardships resulting from the COVID-19 pandemic are well documented (Baddour et al., 2020), significant government funding and assistance, such as federal stimulus checks directed towards lower- and middle-income families, may have alleviated financial impacts of the pandemic (Garner et al., 2020; Kochhar and Sechopoulos, 2020).

This study has several limitations. First, although our sample was large, the survey participants were cancer patients recruited from two NCI-designated cancer centers. Also, the sample was somewhat homogenous regarding race and ethnicity. These sampling biases limit generalizability to more diverse cancer survivors cared for in other settings. Second, the study used ADI, secondary geospatial data, to assess SES and, therefore, may not have fully captured the actual socioeconomic adversity of survivors. Furthermore, information on the cancer treatment status of participants was not collected. These factors may influence the results and lead to more nuanced findings. Third, although the COVID-PPE questionnaire was developed by experts in psycho-oncology and psychometrically evaluated with over 10,000 cancer survivors, its concurrent validity has yet to be demonstrated. Last, the survey was conducted during the initial phases of the COVID-19 pandemic, potentially limiting generalizability to the later phases of the pandemic. Future work should evaluate long-term impacts of the COVID-19 pandemic across multiple psychosocial risks. Notwithstanding these limitations, our study suggests that negative COVID-19-related experiences put cancer survivors at greater risk for financial burden and disruption to daily activities and social interaction, which can have downstream effects on HRQoL and psychological distress. These negative effects were buffered by stress management skills and social support. Financial navigation, such as financial and legal counseling, and other types of psychological and social work interventions, including positive reappraisal or reframing and recommendation to maintain social contacts through social media or video calls, may be useful to mitigate financial burden and disruption to social interaction due to the pandemic.

### 4.1 Conclusion

In conclusion, greater negative COVID-19-related experiences were associated with higher depression and poor HRQoL. Financial hardship, disruption to daily activities and social interaction mediated the association between negative COVID-19-related experiences and anxiety, depression, and HRQoL, and perceived stress management skills and social support buffered these relationships. Augmentation of cancer support and financial counseling services may help cancer patients mitigate the adverse effects of major disruptive events and protect their well-being.

## Data Availability

The raw data supporting the conclusions of this article will be made available by the authors, without undue reservation.
