# Supplementary material for: The impact of negative COVID-19 experiences on cancer survivors’ health-related quality of life and psychological distress: a moderated mediation model
Source: Front Psychol. 2024 Sep 12;15:1423106. doi: 10.3389/fpsyg.2024.1423106 (PMC11440988; doi:10.3389/fpsyg.2024.1423106)
Supplement: Supplementary file 1 [file Data_Sheet_1.docx]

**Supplementary Materials**

**Table S1. Sample Sociodemographic, Medical, and Psychosocial Characteristics**

|  | **Mean**  **(or N)** | **SD**  **(or %)** |
| --- | --- | --- |
| **Sociodemographic Characteristics** | | |
| **Age** | 63.80 | 12.30 |
| **Recruitment Site** |  |  |
| MDACC | 9256 | 95.90 |
| SCCC | 395 | 4.09 |
| **Female** | 5534 | 57.30 |
| **Race/Ethnicity** |  |  |
| Non-Hispanic White | 7942 | 82.30 |
| Non-Hispanic Black | 371 | 3.84 |
| Non-Hispanic, all other races | 274 | 2.84 |
| Hispanic, any race | 897 | 9.29 |
| Refused/Missing | 167 | 1.73 |
| **Area Deprivation Index** | 39.60 | 24.50 |
| **Insurance Type** |  |  |
| Managed Care | 4855 | 50.30 |
| Medicaid | 48 | 0.50 |
| Medicare | 4420 | 45.80 |
| Other/Self-pay | 328 | 3.40 |
| **Medical Characteristics** | | |
| **Cancer Site** |  |  |
| Solid Cancer | 8156 | 84.50 |
| Hematologic Malignancy | 1400 | 14.50 |
| Missing | 95 | 0.98 |
| **Cancer Stage** |  |  |
| Non-metastatic (Stages 0-III) | 7462 | 77.30 |
| Metastatic (Stage IV) | 2189 | 22.70 |
| **Psychosocial Characteristics** | | |
| Adverse COVID-19 Experiences | 7.32 | 1.88 |
| Health-related Quality of Life | 19.00 | 5.30 |
| Depressive Symptoms | 1.75 | 0.98 |
| Anxiety Symptoms | 2.33 | 0.92 |
| Healthcare Disruption | 1.76 | 1.13 |
| Disruption to Daily Activities & Social Interaction | 2.24 | 0.86 |
| Financial Hardship | 1.09 | 0.85 |
| Perceived Stress Management Skills | 2.67 | 0.60 |
| Social Support | 2.60 | 0.63 |

MDACC: University of Texas MD Anderson Cancer Center; SCCC: University of Miami Sylvester Comprehensive Cancer Center.

**Table S2. Parallel Mediation Model of HRQOL**

| **Regression Coefficients** |  |  | | |
| --- | --- | --- | --- | --- |
| **Predictors** | **Outcome** | **Mediators** | | |
|  | **HRQOL** | **HCD** | **DDASI** | **FH** |
| Intercept | **24.839**** | **2.176**** | **2.570**** | **1.641**** |
| **Exposure** |  |  |  |  |
| Negative COVID-19 Experiences | **-0.083*** | **0.005** | **0.043**** | **0.057**** |
| **Covariates** |  |  |  |  |
| Age | **0.023**** | **-0.004*** | **-0.005**** | **-0.010**** |
| Gender, Female | **-0.498**** | **0.102**** | **0.210**** | **0.056*** |
| Race/Ethnicity |  |  |  |  |
| Non-Hispanic Black | 0.212 | **-0.189*** | -0.088 | 0.079 |
| Non-Hispanic, Other race | **0.832*** | **0.151*** | 0.005 | **0.148*** |
| Hispanic | 0.314 | -0.039 | 0.051 | **0.200**** |
| Area Deprivation Index | **-0.007*** | 0.001 | **-0.002**** | **0.002**** |
| Insurance Type |  |  |  |  |
| Managed Care | **0.806*** | **-0.269*** | **-0.108*** | **-0.101*** |
| Medicaid/Medicare | 0.067 | **-0.170*** | -0.054 | -0.090 |
| Cancer Type, Hematologic | **-0.754**** | -0.025 | **0.090*** | 0.000 |
| Cancer Stage, Metastatic | **-1.139**** | 0.001 | 0.021 | 0.007 |
| Study Center, MD Anderson | 0.323 | -0.050 | **-0.308**** | **-0.391**** |
| **Mediators** |  |  |  |  |
| Healthcare Disruption (HCD) | **-0.683**** | - | - | - |
| Disruption to Daily Activities (DDASI) | **-1.798**** | - | - | - |
| Financial Hardship (FH) | **-1.206**** | - | - | - |
| **Total, Direct and Indirect Effects** |  |  |  |  |
|  | **Effect** | **SE** | **LLCI** | **ULCI** |
| **Total Effect (Direct + Indirect)** | **-0.232** | 0.032 | -0.295 | -0.169 |
| **Direct Effect** | **-0.083** | 0.028 | -0.139 | -0.028 |
| **Total Indirect Effect** | **-0.149** | 0.016 | -0.180 | -0.116 |
| Healthcare Disruption | -0.004 | 0.005 | -0.013 | 0.006 |
| Disruption to Daily Activities | **-0.077** | 0.010 | -0.097 | -0.057 |
| Financial Hardship | **-0.068** | 0.0076 | -0.083 | -0.054 |

Note: *p<.05; **p<.001. 95% Bootstrap Confidence Intervals; Reference group: Gender: Male; Race/Ethnicity: Non-Hispanic White; Insurance Type: Other/Self pay; Cancer Type: Solid tumor; Cancer stage: Non-metastatic (Stages 0-III); Study Center: Miami.

**Table S3. Conditional Indirect Effects of the HRQOL moderated mediation model**

|  | **Stress Management Ability (SMA)** | **Social**  **Support (SS)** | **Indirect Effect** | **Standard Error** | **LLCI** | **ULCI** |
| --- | --- | --- | --- | --- | --- | --- |
| **DDASI** |  |  |  |  |  |  |
|  | 2.0631 | 1.9783 | -0.0871 | 0.0118 | -0.1105 | -0.0648 |
|  | 2.0631 | 2.6043 | -0.0929 | 0.0124 | -0.1176 | -0.0694 |
|  | 2.0631 | 3.2304 | -0.0987 | 0.0136 | -0.1265 | -0.073 |
|  | 2.6662 | 1.9783 | -0.0690 | 0.0096 | -0.088 | -0.0509 |
|  | 2.6662 | 2.6043 | -0.0748 | 0.0098 | -0.0943 | -0.0561 |
|  | 2.6662 | 3.2304 | -0.0806 | 0.0108 | -0.1023 | -0.0598 |
|  | 3.2692 | 1.9783 | -0.0509 | 0.0083 | -0.0681 | -0.0355 |
|  | 3.2692 | 2.6043 | -0.0567 | 0.0079 | -0.0725 | -0.0418 |
|  | 3.2692 | 3.2304 | -0.0625 | 0.0087 | -0.0798 | -0.0461 |
| **FH** |  |  |  |  |  |  |
|  | 2.6662 | 1.9783 | -0.0699 | 0.0086 | -0.0871 | -0.0541 |
|  | 2.6662 | 2.6043 | -0.0589 | 0.0068 | -0.0726 | -0.0463 |
|  | 2.6662 | 3.2304 | -0.0479 | 0.0072 | -0.063 | -0.0347 |

Note: 95% bootstrap confidence intervals for the conditional indirect effects of disruption to daily activities and social interaction (DDASI) and financial hardship (FH) at low (16^th^ percentile, SMA= 2.0631; SS=1.9783), medium (50^th^ percentile, SMA=2.662; SS=2.6043), and high (84^th^ percentile, SMA= 3.2692, SS= 3.2304) values of stress management ability and social support.

**Table S4. Parallel Mediation Model of Depressive Symptoms**

| **Regression Coefficients** |  |  | | |
| --- | --- | --- | --- | --- |
| **Predictors** | **Outcome** | **Mediators** | | |
|  | **Depressive Symptoms** | **HCD** | **DDASI** | **FH** |
| Intercept | **0.608**** | **2.153**** | **2.578**** | **1.620**** |
| **Exposure** |  |  |  |  |
| Negative COVID-19 Experiences | 0.009 | 0.007 | **0.045**** | **0.057**** |
| **Covariates** |  |  |  |  |
| Age | **-0.005**** | **-0.004*** | **-0.005**** | **-0.010**** |
| Gender, Female | **0.274**** | **0.103**** | **0.211**** | **0.058*** |
| Race/Ethnicity |  |  |  |  |
| Non-Hispanic Black | **-0.065** | **-0.182*** | -0.092* | 0.078 |
| Non-Hispanic, Other race | -0.078 | **0.162*** | 0.006 | **0.148*** |
| Hispanic | 0.005 | -0.040 | 0.029 | **0.176**** |
| Area Deprivation Index | **-0.002**** | 0.001 | **-0.002**** | **0.002**** |
| Insurance Type |  |  |  |  |
| Managed Care | **-0.058** | **-0.251*** | **-0.103*** | -0.084 |
| Medicaid/Medicare | 0.002 | **-0.156*** | -0.045 | -0.078 |
| Cancer Type, Hematologic | **0.048*** | -0.028 | **0.089*** | 0.003 |
| Cancer Stage, Metastatic | -0.039 | -0.002 | 0.020 | 0.007 |
| Study Center, MD Anderson | **-0.152*** | -0.062 | **-0.328**** | **-0.403**** |
| **Mediators** |  |  |  |  |
| Healthcare Disruption (HCD) | **0.110**** | - | - | - |
| Disruption to Daily Activities (DDASI) | **0.465**** | - | - | - |
| Financial Hardship (FH) | **0.236**** | - | - | - |
| **Total, Direct and Indirect Effects** |  |  |  |  |
|  | **Effect** | **SE** | **LLCI** | **ULCI** |
| **Total Effect (Direct + Indirect)** | **0.044** | 0.006 | 0.033 | 0.056 |
| **Direct Effect** | 0.009 | 0.005 | 0.000 | 0.018 |
| **Total Indirect Effect** | **0.035** | 0.004 | 0.028 | 0.042 |
| Healthcare Disruption | 0.001 | 0.001 | -0.001 | 0.002 |
| Disruption to Daily Activities | **0.021** | 0.002 | 0.016 | 0.026 |
| Financial Hardship | **0.013** | 0.001 | 0.011 | 0.016 |

Note: *p<.05; **p<.001. 95% Bootstrap Confidence Intervals; Reference group: Gender: Male; Race/Ethnicity: Non-Hispanic White; Insurance Type: Other/Self pay; Cancer Type: Solid tumor; Cancer stage: Non-metastatic (Stages 0-III); Study Center: Miami.

**Table S5. Conditional Indirect Effects of the Depressive Symptoms model**

|  | **Stress Management Ability** | **Social**  **Support** | **Indirect Effect** | **Standard Error** | **LLCI** | **ULCI** |
| --- | --- | --- | --- | --- | --- | --- |
| **DDASI** |  |  |  |  |  |  |
|  | 2.0629 | 2.6028 | 0.5313 | 0.0151 | 0.5017 | 0.5609 |
|  | 2.6648 | 2.6028 | 0.4594 | 0.0106 | 0.4385 | 0.4803 |
|  | 3.2667 | 2.6028 | 0.3875 | 0.0145 | 0.3590 | 0.4160 |
| **FH** |  |  |  |  |  |  |
|  | 2.0629 | 1.9777 | 0.2330 | 0.0158 | 0.202 | 0.2639 |
|  | 2.0629 | 2.6028 | 0.1893 | 0.0147 | 0.1605 | 0.2181 |
|  | 2.0629 | 3.2278 | 0.1456 | 0.0201 | 0.1062 | 0.1850 |
|  | 2.6648 | 1.9777 | 0.2583 | 0.0151 | 0.2288 | 0.2879 |
|  | 2.6648 | 2.6028 | 0.2146 | 0.0106 | 0.1939 | 0.2354 |
|  | 2.6648 | 3.2278 | 0.1710 | 0.0148 | 0.1419 | 0.2000 |
|  | 3.2667 | 1.9777 | 0.2837 | 0.0202 | 0.2440 | 0.3233 |
|  | 3.2667 | 2.6028 | 0.2400 | 0.0146 | 0.2114 | 0.2686 |
|  | 3.2667 | 3.2278 | 0.1963 | 0.0155 | 0.166 | 0.2266 |

Note: 95% bootstrap confidence intervals for the conditional indirect effects of disruption to daily activities and social interaction (DDASI) and financial hardship (FH) at low (16^th^ percentile, SMA= 2.0629; SS=1.9777), medium (50^th^ percentile, SMA=2.6648; SS=2.6028), and high (84^th^ percentile, SMA= 3.2667, SS= 3.2278) values of stress management ability and social support.

**Table S6. Parallel Mediation Model of Anxiety**

| **Regression Coefficients** |  |  | | |
| --- | --- | --- | --- | --- |
| **Predictors** | **Outcome** | **Mediators** | | |
|  | **Anxiety** | **HCD** | **DDASI** | **FH** |
| Intercept | **1.353**** | **2.153**** | **2.578**** | **1.62**** |
| **Exposure** |  |  |  |  |
| Negative COVID-19 Experiences | -0.002 | 0.007 | **0.045**** | **0.057**** |
| **Covariates** |  |  |  |  |
| Age | **-0.005**** | **-0.004*** | **-0.005**** | **-0.010**** |
| Gender, Female | **0.172**** | **0.103**** | **0.211**** | **0.058*** |
| Race/Ethnicity |  |  |  |  |
| Non-Hispanic Black | **0.137*** | **-0.182*** | **-0.092*** | 0.078 |
| Non-Hispanic, Other race | **0.243**** | **0.162*** | 0.006 | **0.148*** |
| Hispanic | **0.305**** | -0.040 | 0.029 | **0.176**** |
| Area Deprivation Index | 0.000 | 0.001 | **-0.002**** | **0.002**** |
| Insurance Type |  |  |  |  |
| Managed Care | 0.054 | **-0.251*** | **-0.103*** | -0.084 |
| Medicaid/Medicare | **0.102*** | **-0.156*** | -0.045 | -0.078 |
| Cancer Type, Hematologic | **0.131**** | -0.028 | **0.088*** | 0.003 |
| Cancer Stage, Metastatic | **0.043*** | -0.002 | 0.020 | 0.007 |
| Study Center, MD Anderson | -0.084 | -0.062 | **-0.328**** | **-0.403**** |
| **Mediators** |  |  |  |  |
| Healthcare Disruption (HCD) | **0.130**** | - | - | - |
| Disruption to Daily Activities (DDASI) | **0.353**** | - | - | - |
| Financial Hardship (FH) | **0.103**** | - | - | - |
| **Total, Direct and Indirect Effects** |  |  |  |  |
|  | **Effect** | **SE** | **LLCI** | **ULCI** |
| **Total Effect (Direct + Indirect)** | **0.021** | 0.006 | 0.010 | 0.032 |
| **Direct Effect** | -0.002 | 0.005 | -0.011 | 0.008 |
| **Total Indirect Effect** | **0.023** | 0.003 | 0.018 | 0.028 |
| Healthcare Disruption | 0.001 | 0.001 | -0.001 | 0.003 |
| Disruption to Daily Activities | **0.016** | 0.002 | 0.012 | 0.020 |
| Financial Hardship | **0.006** | 0.001 | 0.004 | 0.008 |

Note: *p<.05; **p<.001. 95% Bootstrap Confidence Intervals; Reference group: Gender: Male; Race/Ethnicity: Non-Hispanic White; Insurance Type: Other/Self pay; Cancer Type: Solid tumor; Cancer stage: Non-metastatic (Stages 0-III); Study Center: Miami.

**Table S7. Conditional Indirect Effects of the Anxiety model**

|  | **Stress Management Ability** | **Social**  **Support** | **Indirect Effect** | **Standard Error** | **LLCI** | **ULCI** |
| --- | --- | --- | --- | --- | --- | --- |
| **DDASI** |  |  |  |  |  |  |
|  | 2.0629 | 2.6027 | 0.0174 | 0.0022 | 0.013 | 0.0215 |
|  | 2.6649 | 2.6027 | 0.0156 | 0.0019 | 0.0118 | 0.0194 |
|  | 3.2668 | 2.6027 | 0.0139 | 0.0018 | 0.0104 | 0.0176 |

Note: 95% bootstrap confidence intervals for the conditional indirect effects of disruption to daily activities and social interaction (DDASI) at low (16^th^ percentile, SMA= 2.0629), medium (50^th^ percentile, SMA=2.6648), and high (84^th^ percentile, SMA= 3.2667) values of stress management ability.

**Figure S1. The conditional indirect effects of disruption to daily activities and social interaction (a) and financial hardship (b) on HRQOL as a function of stress management ability and social support.**

**
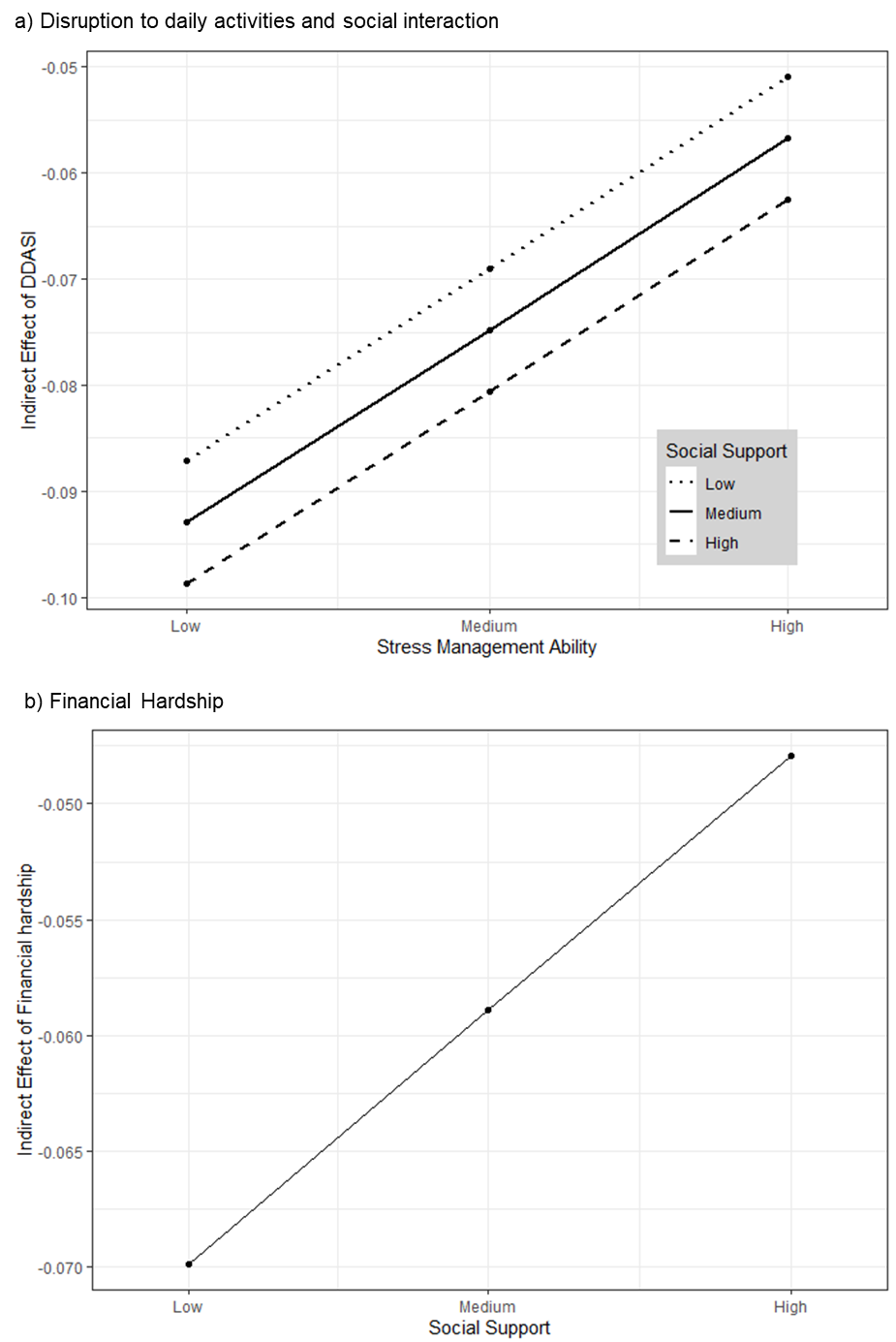
**

**Figure S2. The conditional indirect effects of disruption to daily activities and social interaction (a) and financial hardship (b) on depressive symptoms as a function of stress management ability and social support.**

**
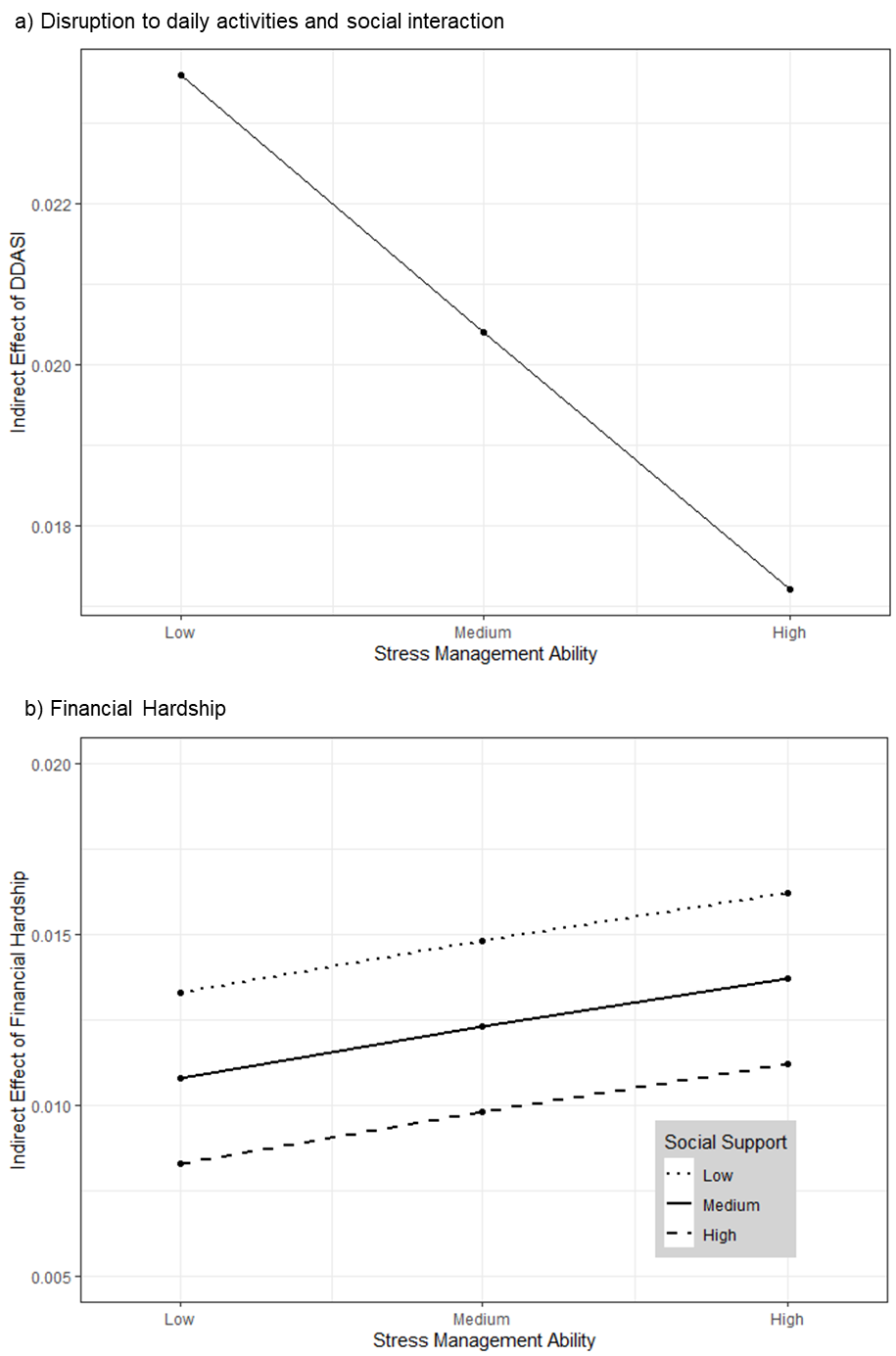
**

**Figure S3. The conditional indirect effect of disruption to daily activities and social interaction on anxiety symptoms as a function of stress management ability.**

**
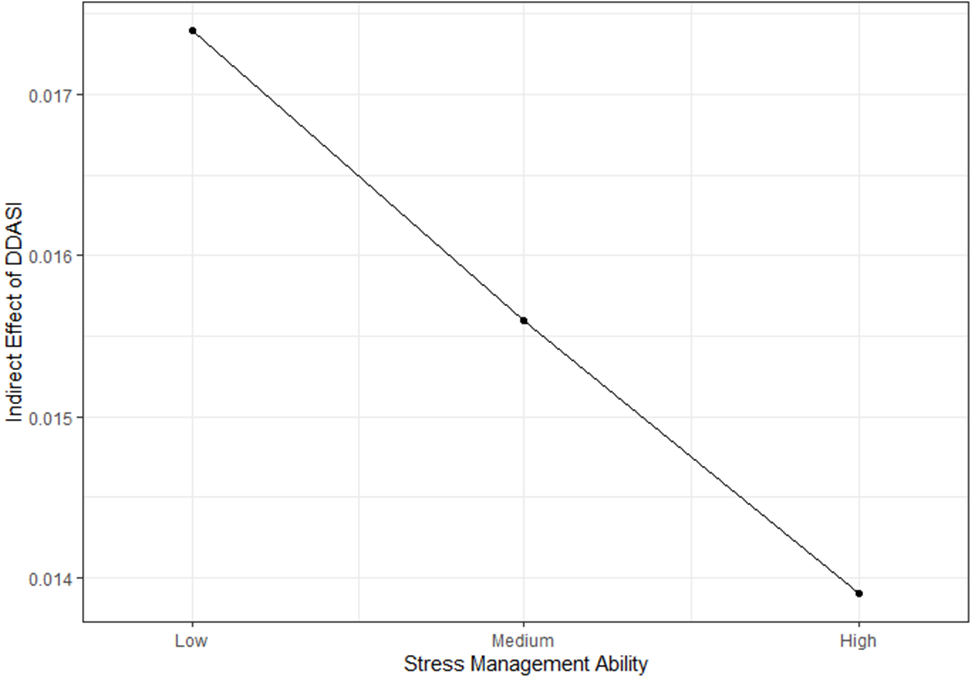
**

**Table S8 Negative COVID-19-related experiences and COVID-19-related Psychosocial and Practical Experience questionnaires.**

| 1. **Negative COVID-19-related experience questionnaire**   **Please answer the questions below to the best of your knowledge. If the item is not applicable, please select N/A. If you do not know the answer, please select D/K**. | |
| --- | --- |
| Questions | Response options |
| 1. To your knowledge, have you been exposed to someone with COVID-19? | Yes  No  D/K |
| 2. Have you been tested for COVID-19? | Yes  No  D/K |
| 1. How many days ago were you tested? | __Days |
| 1. If tested, was your result positive: | Yes  No  D/K |
| 1. If positive, are you currently experiencing COVID-19 symptoms? | Yes  No  D/K |
| 3. If you tested positive for COVID-19, were you hospitalized? | Yes  No  N/A |
| 1. If you were hospitalized, how many nights were you in the hospital? | __Nights  N/A |
| 4. Did a family member or a member of your household test positive for COVID-19? | Yes  No  D/K |
| 1. If yes, how many? | **_** |
| 5. Did a family member or a member of your household die of COVID-19? | Yes  No |
| 1. If yes, did they have COVID-19 symptoms (e.g., fever, cough)? | Yes  No |
| 6. Were any friends, co-workers or neighbors diagnosed with COVID-19 | Yes  No |
| 1. If yes, how many? |  |
| 7. Did a friend, co-worker or neighbor die of COVID-19? | Yes  No  D/K |
| 1. If yes, how many? |  |
| 8. If you practiced social isolation/stay at home/quarantine, for how many days  did it last (total number of days up to today if still practicing isolation)? | **__**Days |
| 9. Do you have any of the following risk factors or experienced symptoms associated with COVID-19: |  |
| 1. ≥ 60 years of age | Yes  No |
| 1. Comorbidities such as diabetes, hypertension, kidney disease, and/or respiratory illnesses (e.g., COPD, asthma) | Yes  No |
| 1. International travel or travel to COVID-19 hotspots | Yes  No |
| 1. Exposure to someone who tested positive to COVID-19 | Yes  No |
| 1. Visiting/working in a nursing home or hospital | Yes  No |
| 1. Fever | Yes  No |
| 1. Dry cough | Yes  No |
| 1. Shortness of breath | Yes  No  N/A |
| 10. Did you lose your job or primary source of income due to COVID-19? | Yes  No  N/A |
| 11. Did your spouse or partner lose their job or primary source of income? | Yes  No  N/A |
| 12. If employed, are you currently: | Working from home  Commuting to work  N/A |
| 13. Due to COVID-19, my household income has: | Decreased  Increased  Not changed |
| 1. If your income decreased, what was the reason (check as many as apply): | Lost job  Spouse/Partner lost job  Assisting family.  Inability to work at home.  Other |
| 1. If your income increased, what was the reason (check as many as apply): | Started a new job  Spouse/Partner started a new job  My work became busier  Other |
| 14. How often are you spending time outside your home? | No time  Once a week  Every 2-3 days  Normal routine |
| 15. Are you accomplishing more or less (e.g., activities, tasks, hobbies, interests)? | More  Less  Same |
| 16. Due to COVID-19, did you decide not to: |  |
| - 1. Attend a scheduled in-person **general medical appointment** not cancelled due to COVID-19? | Yes  No |
| - 1. Attend a scheduled in-person **cancer appointment or treatment** not cancelled due to COVID-19 | Yes  No |
| - 1. Seek **emergency care** in an urgent care facility or emergency room? | Yes  No |
| 17. Did you participate in a Telehealth **medical appointment** (e.g., Zoom, Facetime) since COVID-19 pandemic? | Yes  No |
| 1. If yes, how many? |  |
| 1. If yes, how many were for **cancer care**? |  |
| 1. How many were for other **medical care**? |  |
| 18. If you had a Telehealth appointment for **cancer care**, how satisfied are you with your experience? | Very dissatisfied  Somewhat dissatisfied  Neutral  Somewhat Satisfied  Very Satisfied |
| 19. If you had a Telehealth appointment for **general care**, how satisfied are you with your experience? | Very dissatisfied  Somewhat dissatisfied  Neutral  Somewhat Satisfied  Very Satisfied |
| 1. **COVID-19-related Psycchosocial and Practical Experiences questionnaire (COVID-PPE)**   **Please indicate the extent to which you agree or disagree with the following statements. Please use the scale below:**  **0 = Strongly Disagree 1 = Disagree 2 = Neither agree or Disagree 3 = Agree 4 = Strongly Agree** | |
| **COVID-19 Specific Distress (Emotional and Physical Reactions):**  **Since the breakout of the COVID-19 pandemic:** | |
| 1. I feel anxious about getting COVID-19 *(or if positive: I am anxious about becoming ill)*. | |
| 1. I worry about possibly infecting others. | |
| 1. I am concerned about a family member or close friend getting or dying from COVID-19. | |
| 1. I worry about the possibility of dying from COVID-19. | |
| 1. I fear how the COVID-19 pandemic will impact my cancer care or recovery. | |
| 1. I am concerned that cancer puts me at greater risk for being infected or dying from COVID-19. | |
| 1. I feel I have no control over how COVID-19 will impact my life. | |
| 1. I have experienced feelings of sadness or depression. | |
| 1. I feel negative and/or anxious about the future. | |
| 1. I have experienced changes in my sleep. | |
| 1. I have experienced changes in my eating. | |
| 1. I have experienced difficulty concentrating. | |
| 1. I have experienced feelings of social isolation or loneliness. | |
| **Health Care Disruptions and Concerns (Concerns About Medical Care):**  **Since the breakout of the COVID-19 pandemic:** | |
| 1. My general medical care has been disrupted or delayed. | |
| 1. My cancer care or follow-up has been disrupted or delayed. | |
| 1. My healthcare providers have taken the necessary measures to address COVID-19. | |
| 1. I received adequate information on prevention, protection or care for COVID-19 from my cancer care providers. | |
| **Disruption to Daily Activities and Social Interactions:**  **Since the breakout of the COVID-19 pandemic:** | |
| 1. I have experienced disruptions in day to day social interactions with family and/or friends. | |
| 1. I have not been able to adequately take care of family members or friends I provide for. | |
| 1. I have been unable to perform my typical daily routines (e.g., work, physical activity, leisure activity). | |
| 1. I have experienced conflict with household members (e.g., spouse/partner, children, parents, others). | |
| 1. I have had difficulty or been unable to perform my work as usual. | |
| 1. I have had difficulty taking care of my children’s needs (e.g., providing care, supervising schoolwork) and/or balancing their needs with other responsibilities. | |
| **Financial Hardship:**  **Since the breakout of the COVID-19 pandemic:** | |
| 1. I have experienced financial difficulties. | |
| 1. I have not been able to purchase or obtain basic necessities (e.g., food, personal care products). | |
| 1. I have been anxious about losing or having lost my job, or my primary source of income. | |
| 1. I have not been able to adequately provide for others I financially support. | |
| 1. I feel anxious about being able to maintain or not having adequate health care insurance. | |
| **Perceived Benefits:**  **Since the breakout of the COVID-19 pandemic:** | |
| 1. I have greater appreciation for my family and close friends. 2. I have deeper appreciation for life. | |
| 1. I have been more grateful for each day | |
| 1. I have been more accepting of things I cannot change. | |
| 1. I have found new ways of connecting with family and friends. | |
| 1. I have used my experience in coping with cancer to deal with COVID-19. | |
| **Functional Social Support:**  **Since the breakout of the COVID-19 pandemic:** | |
| 1. I have received emotional support from family or friends when needed. | |
| 1. I have received tangible support (e.g., financial, practical) from family or friends when needed. | |
| 1. I am (or “have been”) there to listen to other’s problems when needed. | |
| 1. I have helped others with financial or practical support. | |
| **Perceived Stress Management (Ability to Manage Stress):**  **Since the breakout of the COVID-19 pandemic:** | |
| 1. I am able to recognize thoughts and situations that make me feel stressed or upset about COVID-19. | |
| 1. I am able to practice relaxation (e.g., deep breathing, meditation) when feeling stress about COVID-19. | |
| 1. I am able to seek information and plan accordingly to address concerns over the COVID-19 pandemic. | |
| 1. I can re-examine negative thoughts and gain a new perspective when concerned about COVID-19. | |
| 1. I can give myself the caring and tenderness I need. | |

**Reference:**

Saez-Clarke E, Otto AK, Prinsloo S, et al. Development and initial psychometric evaluation of a COVID-related psychosocial experiences questionnaire for cancer survivors. Quality of life research. 2023 Dec;32(12):3475-3494. DOI 10.1007/s11136-023-03456-4. 
